# Fungal Endophytes Are Associated with Improved Salinity Responses Across Quinoa Ecotypes

**DOI:** 10.3390/plants15152356

**Published:** 2026-07-30

**Authors:** Roberto Miño, Gabriel I. Ballesteros, Bárbara E. Valenzuela-Hormazábal, Ricardo Cabeza, Karina B. Ruiz, Karen Balboa-Silva, Marco A. Molina-Montenegro

**Affiliations:** 1 Centro de Ecología Integrativa, Instituto de Ciencias Biológicas, Universidad de Talca, Talca 3464336, Chile; roberto.mino@utalca.cl (R.M.); barbara.valenzuelah@utalca.cl (B.E.V.-H.); marco.molina@utalca.cl (M.A.M.-M.); 2 Dirección de Investigación, Vicerrectoría Académica, Universidad de Talca, Talca 3464336, Chile; 3 Laboratorio de Nutrición Vegetal, Departamento de Producción Agrícola, Facultad de Ciencias Agrarias, Universidad de Talca, Talca 3464336, Chile; rcabeza@utalca.cl; 4 Química y Farmacia, Facultad de Ciencias de la Salud, Universidad Arturo Prat, Iquique 1110939, Chile; karuiz@unap.cl; 5 Departamento de Ciencias Básicas, Facultad de Ciencias, Universidad Santo Tomás, Talca 3473620, Chile; kbalboa@santotomas.cl; 6 Centro de Investigación en Estudios Avanzados del Maule (CIEAM), Universidad Católica del Maule, Talca 3480112, Chile

**Keywords:** fungal endophytes, induced and constitutive tolerance, salinity, *Chenopodium quinoa*, salt-responsive genes, ecotypes

## Abstract

Soil salinity is a major constraint on global crop productivity, highlighting the importance of strategies to enhance plant stress tolerance. This study investigated whether root-associated fungal endophytes derived from a salt-tolerant quinoa ecotype could establish associations with a salt-sensitive ecotype and confer systemic physiological and molecular stress-mitigation responses. Fungal endophytes—two *Alternaria* spp. and one *Setophoma* sp.—were isolated from roots of Pandela (salt-tolerant genotype) and inoculated BO75 (salt-sensitive ecotype) under severe salt stress (400 mM NaCl). Physiological (potential photochemical efficiency, survival), biochemical (malondialdehyde, proline), molecular (expression of ion transporter genes *CqSOS1* and *CqNHX1*), and elemental (Na^+^, K^+^, Cl^−^ distribution by μ-XRF) responses were evaluated. In the salt-sensitive BO75 genotype, endophyte inoculation was associated with improved stress performance, evidenced by reduced oxidative damage (lower MDA), higher proline accumulation, and enhanced photochemical efficiency. Furthermore, the reduced expression of *CqSOS1* and *CqNHX1* suggests an improved ionic balance in inoculated plants. Conversely, re-inoculating Pandela yielded negligible effects, suggesting that these endophytes primarily benefit genotypes lacking inherent salt tolerance. These findings indicate that transferring fungal endophytes from halophytic ecotypes can significantly mitigate stress in sensitive genotypes, highlighting their potential for enhancing crop resilience in saline environments.

## 1. Introduction

Edaphic salinity remains a major bottleneck for global food security and agricultural sustainability [1,2]. To overcome these constraints, innovative biotechnological approaches are essential, specifically the targeted modulation of plant–fungal endosymbiosis to bolster salt-tolerance phenotypes. Through their symbiotic relationship, fungal endophytes significantly influence the physiological and biochemical responses of host plants, thus enhancing their capacity to withstand adverse environmental conditions such as salinity [3,4]. This resilience is primarily mediated through the optimization of nutrient acquisition, phytohormonal signaling, or by the activation of stress-response pathways [5]. Endophytes may also build long-term resilience through the enhancement of root architecture and the strengthening of plant defense mechanisms against abiotic stresses [6,7].

Plant tolerance to salt stress relies on the integration of multiple mechanisms. A key biochemical strategy involves the accumulation of compatible osmolytes like proline, which helps to maintain cellular turgor and protects proteins and membranes from damage under saline conditions [8,9]. Plants also mitigate oxidative stress by enhancing the activity of antioxidant enzymes, reducing the damaging effect of reactive oxygen species (ROS; [10]). One indicator of this protective mechanism is the decreased accumulation of malondialdehyde (MDA), a byproduct of lipid peroxidation and a reliable proxy for oxidative damage [11,12]. At molecular levels, ionic homeostasis depends on the coordinated action of Na^+^/H^+^ antiporters and transporters such as *SOS1* (Salt Overly Sensitive 1) and *NHX1* (Na^+^/H^+^ exchanger 1), which control Na^+^ extrusion and vacuolar sequestration, respectively [13,14,15,16]. These transporters synergistically regulate Na^+^ distribution and K^+^ retention across plant tissues, maintaining ionic and osmotic balance [17,18,19]. Since *SOS1* and *NHX1* respond to salt stress and microbial interactions, they represent reliable molecular markers of salt-tolerance regulation [20]. In this context, the ability of plants to maintain Na^+^/K^+^ ratios can be enhanced through association with beneficial microorganisms, particularly fungal endophytes [21,22]. Several studies have shown that endophytes from extreme environments can improve salt tolerance by regulating ion transport, reducing oxidative stress, and enhancing osmotic adjustment mechanisms [4,23,24]. These responses are frequently associated with the modulation of key ion transporters such as *NHX1* and *SOS1*, improved vacuolar Na^+^ compartmentalization, and enhanced control of reactive oxygen species (ROS).

Moreover, some fungal endophytes may contribute to host salt tolerance by facilitating Na^+^ exclusion and ion balance, ultimately enhancing performance under stress conditions [11,25]. These mechanisms can be reflected in physiological and molecular responses such as improved survival, maintenance of photosynthetic efficiency, reduced oxidative damage, osmolyte accumulation, and regulation of ion transporter gene expression [26,27,28]. The transfer of beneficial endophytic fungal consortiums between crops with different stress adaptations has therefore been proposed as a potential strategy to improve crop resilience, as these consortiums may have a synergistic action compared to single inoculations [11,29]. However, evidence of synergistic action of native endophytic consortia across ecotypes remains limited, as local environmental factors and plant developmental stage may influence their effectiveness [30,31]. Hence, understanding how fungal endophytes modulate plant physiology and molecular responses to salinity remains essential to evaluate their potential contribution to crop stress tolerance.

In the context of salt-tolerant crops, *Chenopodium quinoa* (Willd.) is considered a valuable model crop species due to its adaptability to extreme, marginal environments and wide ecotypic variation in stress tolerance [32,33]. Quinoa is a facultative halophyte, and several studies have reported that moderate salinity levels may not represent stressful conditions for this species, with some genotypes maintaining optimal growth and physiological performance under NaCl concentrations between 100 and 200 mM [34,35,36]. Chilean quinoa comprises two genetically distinct ecotypes: *Salares* (salt flats) and Coastal/Lowland types, which differ markedly in salinity tolerance [34,37]. In Coastal/Lowland ecotypes are generally associated with less saline and more humid environments, displaying lower tolerance to severe salinity stress [34].

In addition to these intrinsic mechanisms, quinoa tolerance may also be influenced by its association with endophytic microorganisms [38]. Previous studies have successfully isolated fungal endophytes such as *Penicillium murcianum* and *Talaromyces minioluteus* from quinoa roots grown in salt flats and demonstrated that re-inoculation of these fungi on endophyte-free quinoa plants can improve salinity tolerance by enhancing osmotic adjustment, maintaining photosynthetic efficiency, and reducing oxidative damage [3,39]. However, these findings were limited to the original host genotype, leaving unresolved whether these beneficial effects can be transferred across quinoa ecotypes with contrasting salt tolerance. Therefore, according to the habitat-adapted symbiosis hypothesis, it is plausible that fungal endophytes from plants adapted to extreme environments may confer adaptive advantages and traits to non-adapted hosts [6,40]. Together, these observations support the idea that endophyte-mediated salinity responses depend on both host genotype and environmental context. This highlights the relevance of evaluating cross-ecotype plant–endophyte interactions in quinoa adapted to contrasting salinity conditions.

Hence, this study evaluated whether fungal endophytes isolated from a salt-tolerant Salares quinoa ecotype are associated with improved salinity responses in a salt-sensitive Coastal/Lowland ecotype under severe salinity stress. Based on the habitat-adapted symbiosis hypothesis, we expected that endophytes isolated from the tolerant ecotype would improve salinity responses in the susceptible genotype, whereas endophyte removal would reduce stress tolerance in the native host. As we were interested in assessing the osmoprotective role of endophytes’ inoculation, we compared non-inoculated and inoculated plants growing under salt stress. Therefore, we evaluated key physiological, biochemical, and molecular markers related to photosynthetic efficiency, oxidative stress, osmotic adjustment, ion homeostasis, and expression of the ion transporter genes *CqSOS1* and *CqNHX1*. This approach provides new insights into the role of fungal endophytes in mediating salinity tolerance and adaptative responses across quinoa ecotypes.

## 2. Results

### 2.1. Fungal Isolation and Molecular Identification

**Figure 1:**
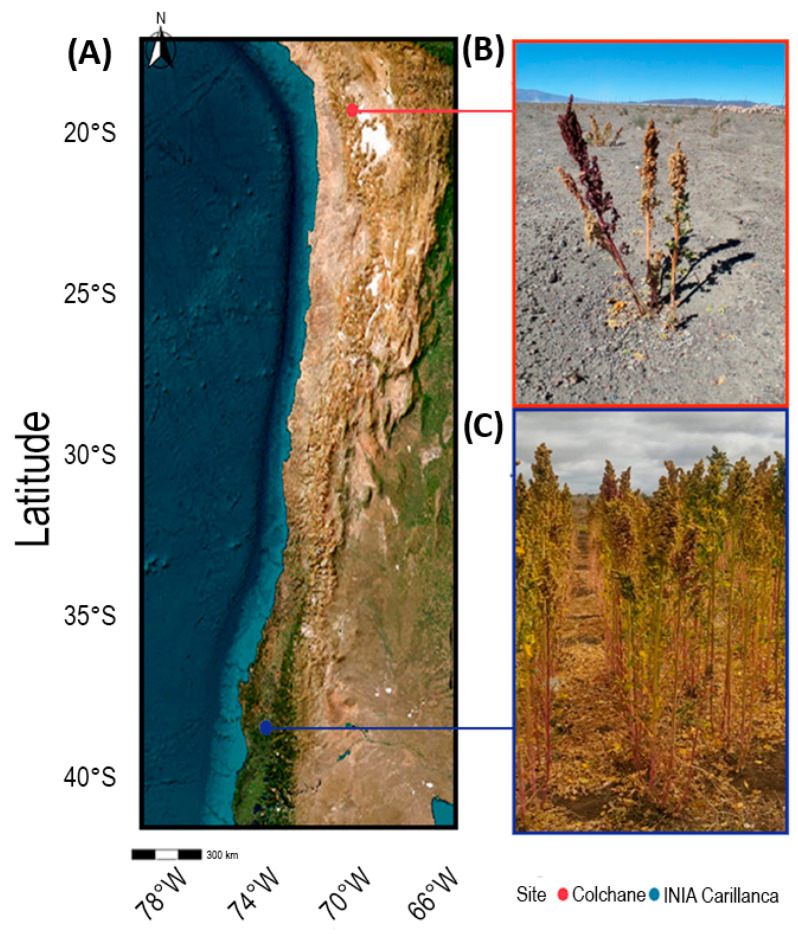
(**A**) Geographic origin of the quinoa ecotypes used in this study: Pandela (Salares ecotype) and BO75 (Coastal ecotype), collected from contrasting environments in Chile (~2600 km apart). (**B**) Mature individuals of the Pandela landrace. Seeds were collected in Colchane (19°16′ S, 68°38′ W), an extremely arid environment characterized by high soil salinity and proximity to the Coipasa salt flats (white area). (**C**) Mature individuals of BO75, a salt-sensitive Coastal ecotype. Seeds were obtained from Vilcún (38°41′ S, 72°25′ W), a rainfed agricultural area characterized by a temperate Mediterranean climate.

From an initial pool of over 100 isolates from Pandela (Figure 1), the ten most prominent isolates were screened for salt tolerance on agar media containing 200 mM NaCl. Following this primary screening, three isolates were selected based on their comparative growth performance under these conditions. PCR amplification (using the universal primers ITS1-ITS4) produced DNA fragments ranging from 550 to 610 base pairs, which were sequenced using a Sanger platform. Comparative BLASTn analysis of the ITS sequences against the UNITE database (Release 19 February 2025; [41]) revealed that, in general, sequence identity exceeded 95% across various sequences from multiple fungal species (Table 1). Isolate #1 (GenBank PX232657) was placed within *Alternaria* section *Embellisia*, sharing maximum identity with *A. chlamydosporigena* (99.817%), *A. embellisia* (99.257%), and *A. tellustris* (99.413%). As unambiguous identification at the species level was not possible, this isolate was conservatively designated as *Alternaria* sp. 1. (section *Embellisia*). Similarly, isolate #2 (GenBank PX232656) was placed within *Alternaria* section *Ulocladioides* due to its high nucleotide similarity to *A. subcucurbitae* (99.77%) and *A. aspera* (99.33%); however, as precise species-level identification could not be achieved, this isolate was designated as *Alternaria* sp. 2. (section *Ulocladioides*). BLASTn analysis of isolate #3 showed a definite match with the reference *Setophoma terrestris* strain (99.8%), while matches to alternative fungal taxa dropped into the 95% to 91% identity range. Hence, and based on this evidence, isolate #3 was provisionally renamed as *Setophoma* spp. 1 (GenBank PX232655).

To further explore the phylogenetic relationships of these isolates, two different maximum likelihood phylogenetic trees were constructed, together with sequences from the two genera (*Alternaria* and *Setophoma*) retrieved from the UNITE FASTA file (Supplementary Figures S1 and S2). This analysis indicated that *Alternaria* sp. 1 groups with different species of the *Alternaria* genus, while *Alternaria* sp. 2 was close to *A. aspera* and *A. elegans*. The phylogenetic analysis also revealed that *Setophoma* sp. 1 is phylogenetically closer to two different strains of *S. terrestris*. Ultrafast bootstrap analysis with 10,000 replications provided robust support for the phylogenetic tree nodes.

### 2.2. Plant Survival

**Figure 2:**
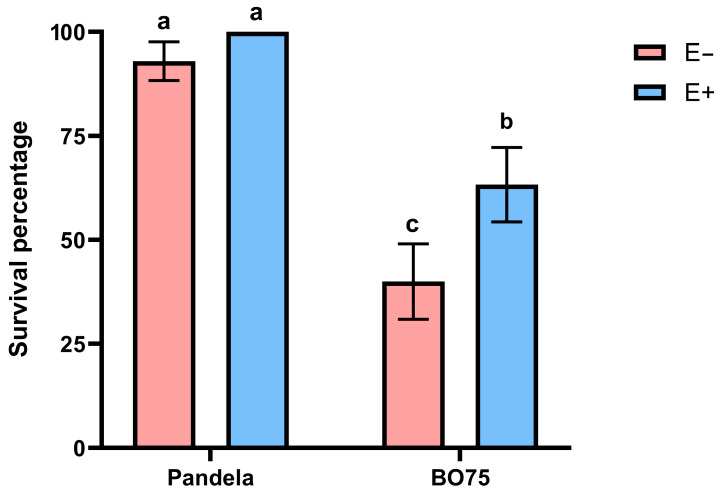
Effects of 400 mM NaCl on survival (percentage) of Pandela (salt-tolerant) and BO75 (salt-sensitive) after 30 days under salt treatment. Sterilized ten-day-old quinoa seedlings were subjected to endophyte inoculation (E^+^) or mock treatment (control, E^−^) at 17 and 22 days after germination. N total = 120 seedlings, divided into four groups with n = 30. Survival was expressed as the percentage of plants that remained alive relative to the initial number of individuals per treatment × 100. Bars are the mean ± standard error. Different letters above the bars represent statistically significant differences (*p* < 0.05) between groups, as determined by two-way factorial ANOVA.

Plant survival under 400 mM NaCl differed between quinoa genotypes and inoculation treatments after 30 days of salt stress (Figure 2). The survival percentages were consistently higher in the Pandela genotype compared to BO75, regardless of inoculation status (*p*-value < 0.0001; Supplementary Table S1). When comparing survival rates between non-inoculated and inoculated Pandela plants, no statistically significant differences were found (E^−^, 93.3% vs. E^+^, 100%; *p*-value = 0.4672). In contrast, inoculated BO75 plants showed significantly higher survival than non-inoculated plants, increasing from 40% to 63.3% (*p*-value = 0.0167). Overall, survival remained higher in Pandela than in BO75 regardless of inoculation status.

### 2.3. Photochemical Efficiency of PSII (Fv/Fm)

**Figure 3:**
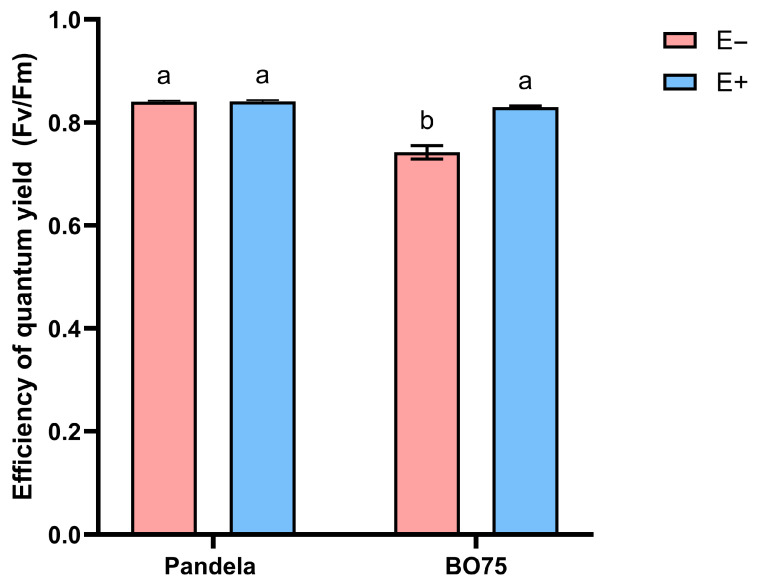
Effects of 400 mM NaCl in the photochemical efficiency of PSII in Pandela (salt-tolerant) and BO75 (salt-sensitive) after 30 days under salt stress. The efficiency quantum yield is the quotient of Fv/Fm, Fv = variable fluorescence of PSII; Fm: maximum fluorescence of PSII. Sterilized ten-day-old seedlings of Pandela and BO75 were subjected to endophyte inoculation (E^+^) or mock treatment (control, E^−^) at 17 and 22 days after germination. Data are the mean of ten biological replicates ± standard error. Different letters above the bars represent statistically significant differences (*p* < 0.05) between groups, as determined by two-way ANOVA.

The photochemical efficiency of Photosystem II (PSII), as indicated by the Fv/Fm ratio, was assessed across different treatment groups (Figure 3). In the Pandela genotype, there were no significant differences in Fv/Fm values between plants with and without endophytes. However, in the BO75 genotype, plants inoculated with endophytes exhibited Fv/Fm ratios similar to those of Pandela, with significantly higher values compared to BO75 plants without endophytes. The results of the two-way factorial ANOVA (Supplementary Table S1) further support this observation, revealing significant main effects of fungal endophyte inoculation (F = 38.92; *p*-value < 0.001) and genotype (F = 34.47; *p*-value < 0.001) on Fv/Fm. Moreover, a significant interaction between fungal inoculation and genotype (F = 23.94; *p*-value < 0.001) was detected, highlighting that the response of BO75 to endophyte inoculation was distinct from that of Pandela.

### 2.4. Lipid Peroxidation

**Figure 4:**
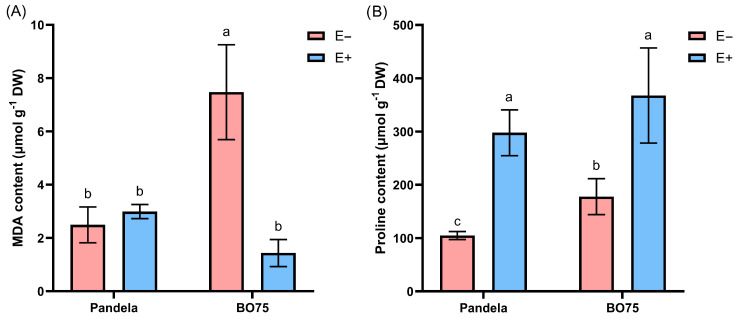
Effects of 400 mM NaCl in the leaf contents of malondialdehyde (MDA) (**A**) and proline (**B**) after 30 days under salt stress. Sterilized ten-day-old seedlings of Pandela (salt-tolerant) and BO75 (salt-sensitive) were subjected to endophyte inoculation (E^+^) or mock treatment (control, E^−^) at 17 and 22 days after germination. Data are the mean of five biological replicates ± standard error. Different letters above the bars represent statistically significant differences (*p* < 0.05) between groups, as determined by two-way ANOVA.

The results revealed significant differences in MDA levels when comparing genotypes, inoculation status (endophytes), and their interaction (Figure 4A; Supplementary Table S1). The MDA levels were notably higher in BO75 plants without endophyte inoculation, indicating greater oxidative stress under salinity. In contrast, BO75 plants inoculated with endophytes exhibited significantly lower MDA levels, suggesting that the presence of endophytic fungi helps mitigate lipid peroxidation under saline conditions. For the Pandela genotype, no significant differences in MDA levels were observed between inoculated and non-inoculated plants, suggesting that this genotype is less affected by salinity stress compared to BO75 (Figure 4A). The two-way factorial ANOVA (Supplementary Table S1) confirmed these results, showing a significant effect of genotype (*p*-value < 0.0014), fungal inoculation (*p*-value < 0.0001), and a significant interaction between fungal inoculation and genotype (F × G; *p*-value < 0.0001).

### 2.5. Proline Content

The results showed that proline content was significantly higher in the presence of endophytes for both genotypes (Figure 4B). In BO75, endophyte-inoculated plants showed significantly higher proline levels than those without endophytes. Similarly, in Pandela, endophyte-inoculated plants also showed an increase in proline content compared to non-inoculated plants, although the difference was less pronounced than in BO75. The two-way factorial ANOVA (Supplementary Table S1) corroborated these observations. Significant main effects were detected for both fungal inoculation (*p*-value < 0.0001) and genotype (*p*-value < 0.0076), indicating that both factors independently influence proline content. However, the interaction between fungal inoculation and genotype (F × G; *p*-value = 0.9459) was not statistically significant.

### 2.6. Expression of SOS1 and NHX1

**Figure 5:**
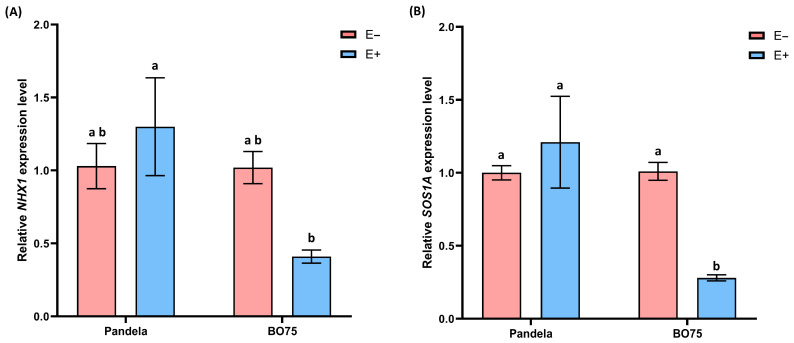
Effects of 400 mM NaCl in the leaf transcript levels of *CqNHX1* (**A**) and *CqSOS1A* (**B**) after 30 days of salt stress. The relative expression was measured in sterilized ten-day-old seedlings of Pandela (salt-tolerant) and BO75 (salt-sensitive) subjected to endophyte inoculation (E^+^) or mock treatment (control, E^−^) at 17 and 22 days after germination. Data are the mean of three biological replicates and three technical replicates ± standard error. Different letters above the bars represent statistically significant differences (*p* < 0.05) between groups, as determined by two-way ANOVA.

The expression of the salt-extrusion genes *CqNHX1* and *CqSOS1A* in *C. quinoa* was significantly influenced by both genotype and fungal endophyte inoculation under salinity stress (Figure 5). In the BO75 genotype, a higher expression level of *CqNHX1* was observed for the E^−^ condition, potentially reflecting increased vacuolar sodium sequestration activity under salt stress (Figure 5A). Conversely, inoculation with endophytes (E^+^) led to a twofold reduction in *CqNHX1* expression. In Pandela, however, no significant differences were observed between treatments. Statistical analysis confirmed significant effects of genotype (*p*-value = 0.0309) and the genotype x endophyte interaction (F × G; *p*-value = 0.034) on *CqNHX1* expression (Supplementary Table S1). Similarly, in BO75, *CqSOS1* expression (Figure 5B) was significantly higher in plants without endophytes compared to inoculated plants, which exhibited significantly lower expression of *CqSOS1A* (*p*-value = 0.0224). In the case of Pandela, no significant differences were observed. Two-way ANOVA revealed significant effects for genotypes (*p*-value = 0.0103) and the F × G interaction (*p*-value = 0.0089) (Supplementary Table S1). Remarkably, expression levels were significantly higher in inoculated Pandela plants compared to inoculated BO75 plants (Figure 5).

### 2.7. Elemental Composition

**Figure 6:**
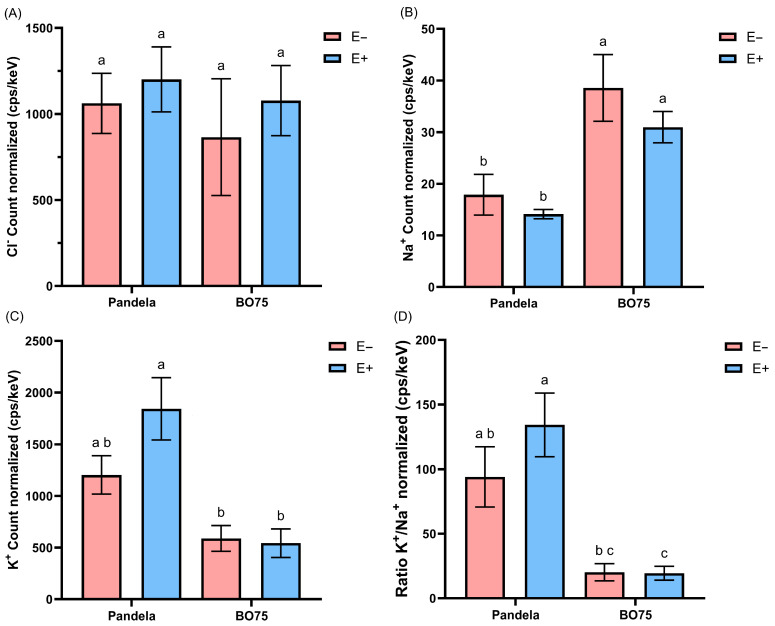
Effects of 400 mM NaCl on leaf elemental composition in Pandela (salt-tolerant) and BO75 (salt-sensitive) quinoa genotypes after 30 days of salt stress. Chlorine (Cl^−^) (**A**), sodium (Na^+^) (**B**), potassium (K^+^) (**C**), and K^+^/Na^+^ ratio (**D**) were measured in 10-day-old sterilized quinoa seedlings subjected to endophyte inoculation (E^+^) or mock treatment (control, E^−^) at 17 and 22 days after germination. Each bar represents the mean ± standard error (n = 12 biological replicates). Different letters above the bars indicate significant differences among groups according to Dunn’s multiple comparison test following a non-parametric Kruskal–Wallis test (*p* < 0.05).

Elemental composition (Na^+^, K^+^, and Cl^−^) measured by μ-XRF on Pandela and BO75 leaf samples showed genotype-dependent responses to endophyte inoculation under saline conditions (Figure 6). Statistical analysis of data using Kruskal–Wallis tests demonstrated that tissue Cl^−^ content (Figure 6A) remained statistically unaffected across groups (χ^2^(3) = 3.96, *p* = 0.2658), suggesting stable Cl^−^ levels irrespective of genotype or endophyte presence. In contrast, significant variations were detected for Na^+^ accumulation (Figure 6B; χ^2^(3) = 23.58, *p* < 0.0001). Pairwise evaluations using Dunn’s test revealed that Na^+^ content was significantly higher in BO75 compared to Pandela, regardless of inoculation status (*p*-adjusted < 0.01), while the presence of the endophyte did not significantly alter Na^+^ levels within genotypes (*p*-adjusted > 0.9999) (Supplementary Table S3). For K^+^ content (Figure 6C), significant overall variations were observed (χ^2^(3) = 17.30, *p* = 0.0006); inoculated Pandela plants (Pandela E^+^) achieved the highest accumulation, being separated from both BO75 E^−^ (*p*-adjusted = 0.0069) and BO75 E^+^ (*p*-adjusted = 0.0026), whereas uninoculated Pandela (E^−^) maintained an intermediate status (Supplementary Table S3). Accordingly, the K^+^/Na^+^ ratio (Figure 6D) varied significantly across groups (χ^2^(3) = 18.55, *p* = 0.0003); the highest homeostatic balance was achieved by the Pandela E^+^ group, outperforming both BO75 E^−^ (*p*-adjusted = 0.0059) and BO75 E^+^ (*p*-adjusted = 0.0030) treatments (Supplementary Table S3). Overall, these results suggest differences in ionic balance and in the spatial distribution patterns of Na^+^, K^+^, and K^+^/Na^+^ ratios between treatments, but not for Cl^−^ (Supplementary Table S2).

## 3. Discussion

In this study, we found that co-inoculation with endophytic fungi (two different *Alternaria* spp. and one *Setophora* sp.) isolated from healthy, asymptomatic tissues from the salt-tolerant quinoa ecotype (Pandela) helps to mitigate some of the negative effects of salt stress in the salt-sensitive ecotype BO75. In contrast, only limited additional benefits were observed when these endophytes were re-introduced into Pandela and for some of the measured parameters. These observations are consistent with previous studies describing context-dependent plant–endophyte interactions in plants adapted to stressful environments [6], where the ecological background in which the host-microbe association evolved influences endophyte effects. Under this framework, endophytes originating from stress-adapted environments may contribute to enhanced stress responses in more susceptible genotypes, such as BO75 [42]. On the other hand, the limited response observed in Pandela may reflect its already high intrinsic tolerance to salinity, potentially reducing the relative contribution of additional endophytic association [42].

Although ITS markers did not allow species-level identification of the isolated endophytes, the sequenced fragments and phylogenetic analysis successfully assigned the endophytes to specific sections within the genera *Alternaria* and *Setophoma*. The genus *Alternaria* is ubiquitous and diverse in terms of their biology, ecology and morphology [43,44]. Isolate #1 (*Alternaria* sp. *1*) was situated within section *Embellisia*, showing high similarity with *A. embellisia* and *A. chlamidosporigena*, a group previously described as endophytes capable of producing bioactive compounds [45]. Based on the sequence analysis, it was found that *Alternaria* spp. 2 is closer to *Alternaria subcucurbitae and A. aspera*; thus, the exact species was not distinguished, as the ITS marker used in this study provided a reliable “genus-level” barcode [46]. While *Alternaria* spp. are mainly regarded as plant pathogenic fungi (producing wilting or decay on the host plant), they have also been reported as one of the most common fungi in various plant species, including halophytes of *Chenopodiacieae,* such as *C. quinoa* [38,47,48]. Moreover, some *Alternaria* species, isolated from different plant species, have been successfully inoculated onto *Nicotiana tabacum* and *Zea mays* and showed plant-growth-promoting activity on these hosts [49,50]. In the case of isolate #3 *(Setophoma* sp. 1), ITS sequencing revealed high phylogenetic proximity to *Setophoma terrestris.* While *S. terrestris* is characterized as the causal agent of “pink root rot” in a wide range of agricultural crops [51], recent studies have identified it as a common endophyte capable of promoting plant growth and improving host health under non-pathogenic conditions [52]. For instance, inoculation with *S. terrestris* has been shown to significantly increase plant height, chlorophyll content, and the accumulation of secondary metabolites like saponins [53]. Overall, our findings support an endophytic lifestyle and a mutualistic interaction for these isolates, as no disease symptoms were observed under the experimental conditions evaluated. This highlights the dynamic nature of the “mutualism-parasitism continuum”, where the ecological outcome of a plant–fungus interaction is not a fixed trait but a plastic state determined by environmental context, host genotype, and interaction stability [42,53,54]. However, ITS sequencing provided insufficient taxonomic resolution to establish potential links between specific species and functional traits [54]. Because functional validation of these relationships was beyond the scope of the present study, future studies should employ multi-locus sequencing using additional markers such as *GAPDH*, *RPB2* and *TEF1*-α to improve taxonomic resolution and clarify the evolutionary drivers of these symbiotic relationships [52].

When comparing the outcomes of endophytic co-inoculation on salt-tolerant and salt-sensitive cultivars under severe saline stress (400 mM NaCl), beneficial responses were observed across several physiological and molecular pathways. These gains are likely driven by a mitigation strategy that enhances the host’s cellular homeostasis and survival capacity [42]. Under these conditions, BO75-inoculated plants showed significantly higher survival than non-inoculated plants, suggesting that the fungal consortium could be associated with improved stress acclimation in the salt-sensitive genotype. Similar improvements in plant survival under saline conditions have been previously reported in plants associated with fungal endophytes, where enhanced tolerance was linked to improved osmotic adjustment, ionic balance, and stress mitigation mechanisms. In contrast, Pandela maintained high survival under both inoculation and control conditions, close to 100% and without significant differences between treatments. These observations are consistent with the inherent salinity tolerance previously described for Salares quinoa ecotypes [34]. The Fv/Fm ratio observed in inoculated plants under salinity is consistent with improved PSII efficiency, suggesting a potential contribution of endophytes to the maintenance of photosynthetic function under stress. Similar responses have been reported in other plant–endophyte systems, where fungal symbionts have been associated with reduced oxidative damage and improved chloroplast stability [55,56]. The significant reduction in malondialdehyde (MDA) levels observed in inoculated BO75 plants is consistent with reduced oxidative damage compared to non-inoculated plants, in agreement with previous studies describing enhanced antioxidant responses in endophyte-associated plants under abiotic stress [5]. In parallel, increased proline accumulation, a well-known osmoprotectant involved in stress responses, highlights the endophytes’ role in biochemical adjustments and stabilization of cellular functions to maintain osmotic balance under saline conditions. Similar increases in proline have been reported in plants inoculated with endophytes under drought or saline stress [8]. The reduced MDA accumulation together with the increased proline observed in inoculated plants suggests improved protection against oxidative damage under salt stress. Proline is known to contribute to osmotic adjustment, ROS buffering, and stabilization of cellular structures during abiotic stress, which may partially explain the lower lipid peroxidation observed in inoculated plants. Together, these responses are consistent with improved redox homeostasis in endophyte-inoculated plants. Previous studies have shown that fungal endophytes may influence antioxidant and ROS-scavenging pathways under abiotic stress, including the modulation of enzymes such as SOD, CAT, and POX [57,58]. Although antioxidant activities were not directly evaluated, the observed physio- and biochemical responses suggest that endophyte inoculation is consistent with improved stress tolerance in the susceptible ecotype under saline conditions. Recent studies highlight the importance of ROS/RNS-mediated signaling and redox balance in coordinating plant response to salinity stress through the integration of antioxidants, osmotic adjustment, and ion homeostasis pathways [59].

At the gene expression level, in BO75, the downregulation of the salt stress-responsive genes *CqNHX1* and *CqSOS1A* following endophyte inoculation under severe salinity may reflect improved ionic balance, rather than an increased requirement for active Na^+^ transport (to either the vacuole or to the extracellular xylem [18]). This pattern is consistent with previous observations in quinoa showing that genotypes with more efficient ion homeostasis do not necessarily require strong induction of sodium transporters to cope with high salinity [60]. In contrast, the higher expression of these genes in non-inoculated BO75 plants may indicate a greater dependence on Na^+^ sequestration and extrusion mechanisms under stress conditions. In Pandela, gene expression remained comparatively stable across treatments, consistent with its inherent salt tolerance and previously reported capacity to maintain ionic balance under high salinity [34,59]. Together, these observations suggest that endophyte inoculation is associated with changes in stress-response gene expression that may contribute to improved stress performance in salt-sensitive genotypes. Notably, the stronger responses observed in BO75 compared with Pandela may indicate that endophyte-associated effects are more detectable in stress-sensitive than already tolerant ecotypes [11]. Because Na^+^ extrusion and compartmentalization are energetically demanding processes under salinity stress, the lower expression of *CqSOS1* and *CqNHX1* in inoculated BO75 plants may indicate a reduced requirement for stress-responsive ion transport activity, consistent with improved ionic balance and lower physiological stress.

Although the specific molecular signaling pathways underlying these responses were not directly investigated, previous studies suggest that endophytic fungi may modulate salinity responses through interconnected ROS, hormonal, calcium-dependent, and ion transport signaling pathways [11,23,59]. Single-cell and spatial transcriptomic approaches may help to better resolve the cellular heterogeneity underlying plant–endophyte responses to salinity stress [61]. These tools may also help to clarify the spatial regulation of ion transport and stress-responsive genes such as *CqSOS1* and *CqNHX1*.

In addition to the mechanisms discussed above, alternative explanations should be considered when interpreting the observed responses. The improved performance of BO75 following endophyte inoculation may not necessarily reflect the direct transfer of adaptive traits from the donor ecotype but could also arise from more general stress buffering effects. For instance, endophytes are known to modulate plant physiology through multiple indirect pathways, including the activation of antioxidant systems, modulation of hormonal signaling, or improved osmotic adjustment [11,62]. Furthermore, the co-inoculation with a consortium of endophytes may influence crop yield, survival and metabolic capability under stress conditions [63,64]. Indeed, such community-level effects have been increasingly recognized as important drivers of plant performance under stress conditions [11,26,65]. Therefore, the responses observed in BO75 are likely the result of complex and potentially interacting mechanisms, rather than a single process of trait transfer. Together, these observations point to a complex interaction between plant physiology and microbial influence under saline conditions.

Endophytes have been frequently associated with improved ion balance in plants’ salinity stress [4,23]. In both genotypes, a reduction in Na^+^ accumulation (although non-significant) was observed for endophyte-inoculated plants. Additionally, in the case of Pandela, an increased K^+^ accumulation and an improvement of the observed K^+^/Na^+^ ratio would be consistent with improved ionic homeostasis under saline conditions when inoculated with endophytes. These responses may be related to the regulation of ion transport processes, which are known to play a role in preventing ion toxicity and maintaining cellular homeostasis under high salinity conditions [17].

μ-XRF mapping further indicated differences in Na^+^ and K^+^ spatial distribution between treatments, providing additional, although indirect, evidence of altered ionic partitioning. While μ-XRF offers spatially resolved insights into elemental distribution, its semi-quantitative nature requires that these observations be interpreted cautiously and in conjunction with physiological and molecular indicators rather than as absolute measures [66]. Despite this, and taken together, these observations are consistent with improved ionic balance in endophyte-inoculated plants, in agreement with previous reports describing beneficial plant–endophyte interactions under saline conditions [5,6,21]. As commonly reported in plant–endophyte studies, these responses are interpreted based on inoculation effects and associated physiological changes, without direct quantification of endophyte persistence over time [6,62]. Further studies integrating quantitative ion analysis and direct assessment of endophyte colonization would help to refine the interpretation of these responses and clarify the underlying mechanisms.

The responses observed following co-inoculation highlight the context-dependent nature of plant–microbe interactions and their potential contribution to improved plant performance under salinity stress. These findings are consistent with the growing body of evidence showing that microbial roles are strongly influenced by environmental conditions and host-specific factors [65,67]. In this context, environmental stressors, such as salinity, may influence microbial behavior, sometimes shifting interactions from pathogenic or neutral to beneficial outcomes [68]. Similar context-dependent responses have been described across different plant–microbe systems [62,69], suggesting that under saline environments some microorganisms may contribute to plant stress responses (through a ‘rescue effect’) rather than acting solely as pathogens. Overall, these findings are consistent with recent reports describing the contribution of fungal endophytes to salinity tolerance through coordinated physiological and ionic responses.

### Limitations and Concluding Remarks

Quinoa, due to its ecotypic differentiation and ability to grow under marginal and sub-optimal conditions, represents a valuable model for studying plant adaptation to salinity stress [32]. In this context, our findings suggest that co-inoculation with selected endophytic fungal strains may contribute to enhanced stress responses in susceptible genotypes, highlighting their potential relevance in plant–microbe interactions under saline environments.

The present study evaluated plant responses to a simplified fungal consortium derived from a salt-tolerant quinoa ecotype under severe salinity conditions. The selected isolates represent only a fraction of the culturable endophytic microbiome and were identified at the *genus* level using ITS markers; therefore, future studies should consider a multi-locus sequencing approach to provide the phylogenetic depth for definitive species identification [54]. Nevertheless, the coordinated physiological, biochemical, and molecular responses observed after inoculation are consistent with biologically relevant plant–endophyte interactions. Because a mixed consortium was used, the contribution of individual isolates remains unresolved. Future studies using single-strain and controlled co-inoculation approaches will help to clarify whether these effects are driven by specific isolates or by *consortium*-associated interactions. In addition, benomyl and rifampicin treatments were applied uniformly prior to inoculation [70]. However, potential interactions between these compounds and plant stress responses under high salinity deserve further evaluation. The experimental design focused specifically on plant performance under severe salinity conditions. This approach considered the halophytic trait of quinoa and the ecological relevance of saline environments for this species [34,35,36]. In agreement with this rationale, Miño et al. [41] reported limited effects of quinoa-associated endophytes under low or absent salinity conditions. Quantitative evaluation will be necessary to assess the persistence, stability, long-term colonization dynamics, and biosafety of fungal endophyte *consortiums* under several environmental conditions.

Despite these limitations, these results support the idea that fungal endophytes may represent promising biological components for enhancing plant performance under stress conditions [10,11]. Future studies integrating quantitative ion analyses and omics-based approaches will help refine the mechanistic understanding of these interactions. Concomitantly, the practical application of endophytic communities as biotechnological tools requires further validation to determine if these associations influence key agronomic traits such as grain nutritional quality and yield. Furthermore, the long-term stability of plant–endophyte interactions should be further evaluated, particularly as productive ecosystems are expected to become increasingly arid and saline in the coming decades.

## 4. Materials and Methods

### 4.1. Fungal Isolation

For fungal isolation, a total of ten *C. quinoa* plants belonging to the Pandela genotype were randomly selected for root sampling from a plantation in Cariquima, Colchane, Chile (19°16′ S, 68°38′ W). Plants were carefully transported to the laboratory under controlled conditions to maintain their integrity and ensure optimal preservation. The roots were gently rinsed to remove any adhering rhizosphere material. Then, they were surface sterilized by sequential immersion in 70% ethanol for 2 min, followed by 2.5% sodium hypochlorite for 1 min, and a final rinse in sterile distilled water for 2 min [71]. After sterilization, the roots were cut into approximately 1 cm segments. These segments were transferred to potato dextrose agar (PDA) plates supplemented with 50 mg/L chloramphenicol to prevent bacterial growth. Then, to promote the isolation of salt-tolerant fungal endophytes, the ten most prevalent strains were cultured in PDA plates supplemented with 200 mM NaCl to simulate a moderately saline environment (mild salinity stress); this salt concentration is well within the tolerance range where *C. quinoa* can still exhibit productive growth while encompassing the range of salinity observed in soils where C. quinoa is produced [72]. Then, plates were incubated at 28 °C in the dark for 7 to 14 days, with daily monitoring for fungal growth. From this collection, three representative culturable fungal isolates were selected for downstream analyses. Selection criteria included: (i) successful isolation from quinoa roots; (ii) stable growth under laboratory conditions; (iii) morphological differences during culture; and (iv) the absence of visible inhibitory interactions among isolates. To obtain pure cultures, emerging colonies were transferred onto fresh PDA plates.

### 4.2. Fungal Molecular Identification

For molecular identification, fungal isolates were grown on potato dextrose agar (PDA) plates, and mycelium was collected directly from actively growing colonies. Total genomic DNA was extracted using a CTAB-based protocol [73]. Then, PCR was conducted to amplify the ribosomal RNA internal transcribed spacer (ITS) region using the universal primers ITS1 and ITS4. Resulting amplicons were sequenced using Sanger sequencing technology with the aforementioned ITS primers on both strands. Electropherograms were analyzed using Chroma software v2.6.6 for quality assessment of each strand, which was conducted by visual inspection and trimming low-quality or unreadable areas. After trimming, taxonomic assignment was conducted by aligning each nucleotide sequence with a reference FASTA UNITE database (eukaryotic nuclear ribosomal ITS region [40]), using the local BLASTn package (2.12.0+) with default parameters. Trimmed sequences were deposited in NCBI GenBank (accession numbers PX232655–PX232657). ITS-based analyses were used primarily for genus-level ID, as species-level resolution remained limited for the *Alternaria* taxa. To assess the phylogenetic position of the fungal isolates, multiple sequence alignments were created with CLUSTAL Omega (EMBL-EBI), using as reference sequences from the identified genera retrieved from UNITE. These alignments were then subjected to phylogenetic inferences with the maximum likelihood inference method, using IQ-tree v3.0.1 and Modelfinder to determine best-fit models. Ultrafast bootstrap analysis [74] was performed with 10,000 replicates to assess node support. Finally, generated trees were displayed in iTOL v7 and were rooted with either *Alternaria longipes* or *Pseudopyrenochaeta lycopersici* as outgroups.

### 4.3. Plant Material and Growth Conditions

To evaluate the effects of fungal endophytes on salinity responses in contrasting quinoa ecotypes, two *Chenopodium quinoa* genotypes differing in salinity tolerance were used: Pandela (salt-tolerant Salares ecotype [34]) and BO75 (salt-sensitive Coastal ecotype) (Figure 1). The two ecotypes originate from geographically distant environments separated by approximately 2600 km. Pandela seeds were collected from plants growing in their habitat, whereas BO75 seeds were provided by the germplasm bank of the Agricultural Research Institute of Chile (INIA Carillanca, Vilcún, Temuco, Chile). Sterilized seeds from both genotypes were sown in 10 L pots containing a sterile substrate mixture of peat, perlite, and vermiculite (2:2:1) [41]. Plants were maintained under controlled greenhouse conditions (26 °C/15 °C day/night, 1400 µmol m^−2^ s^−1^ PAR, and 70% relative humidity). Pots were separated by 1 m to minimize plant–plant interactions and were rotated weekly to homogenize micro-environmental conditions within the greenhouse.

### 4.4. Endophyte Reduction Treatment

Benomyl and rifampicin treatments were used to reduce endogenous fungal and bacterial loads and generate endophyte-reduced plant material prior to inoculation [41]. Two-week-old seedlings of both genotypes were treated with an antifungal agent, Benlate^®^ (benomyl [methyl [1-butylamino carbonyl]-1H-benzimidazol-2-yl] carbamate (DuPont, Wilmington, DE, USA)), applied to both the substrate and the foliar parts of the plants. All seedlings were also treated with a broad-spectrum antibiotic, rifampicin (50 μg mL^−1^), to eliminate endophytic bacteria [41]. To verify the efficacy of the antibiotic and antifungal treatments, four seedlings from each genotype were randomly selected after five days of antifungal and antibiotic application for root sampling; a subset of root segments were plated on Luria–Bertani (LB) agar and potato dextrose agar (PDA) to detect bacterial and fungal growth, respectively. LB plates were incubated at 28 °C for five days, and PDA plates at 28 °C for two weeks.

Additional root segments were cleared in 10% KOH (*w*/*v*), stained with trypan blue (0.4%) in an acid glycerol solution, and examined by light microscopy at 400× magnification (Motic BA410) to verify the absence of fungal structures. Plants showing no detectable microbial growth nor fungal structures were considered endophyte-reduced (E^−^).

### 4.5. Salinity Treatment and Colonization Assessment

Salinity treatment was applied by irrigating all plants every two days with a 400 mM NaCl solution for 30 days. This salinity level (400 mM NaCl) was selected to impose severe saline stress and to maximize physiological differences between contrasting quinoa ecotypes, consistent with previous quinoa studies [34,36]. Irrigation was applied to field capacity. The high porosity and low water-holding capacity of the peat–perlite–vermiculite substrate ensured adequate drainage and prevented waterlogging.

Thirty days after the last inoculation, three plants per treatment were randomly selected for root sampling to evaluate fungal colonization using plating and microscopy approaches as aforementioned. Colonization was considered positive when fungal structures were observed within root tissues. However, it should be noted that a quantitative assessment of colonization efficiency (e.g., via qPCR) was not performed in this study; fungal presence was evaluated qualitatively to verify the establishment of the symbiosis prior to stress exposure.

### 4.6. Physiological and Biochemical Markers

#### 4.6.1. Plant Final Survival

For each experimental group (endophyte presence/genotype; N = 30), plant survival was recorded at the end of the experiment (30 days) as a binary parameter, considering that the plant was dead if it presented more than 90% of damaged tissue [75]. Survival was expressed as the percentage of plants that remained alive (S) relative to the initial number of individuals per treatment (30 plants) × 100.

#### 4.6.2. Photochemical Efficiency of PSII (Fv/Fm) Measurements

To measure the photochemical efficiency of Photosystem II (PSII), chlorophyll fluorescence parameters were assessed for each group of quinoa plants using a pulse-amplitude modulated fluorometer (FMS 2, Hansatech Instruments Ltd., Norfolk, UK) following the protocol established by Maxwell and Johnson [76]. For each experimental condition, 10 plants were evaluated, and three independent measurements were taken per plant. Prior to measurements, leaves were dark-adapted for 30 min to ensure optimal photochemical efficiency. The maximum quantum yield of PSII (Fv/Fm) was calculated by the formula of variable fluorescence (Fv) = Fm − Fo, where Fm is the maximum fluorescence yield and Fo is the minimum fluorescence yield.

#### 4.6.3. Lipid Peroxidation Measurements

Assessment of lipid peroxidation was conducted by measuring malondialdehyde (MDA) concentration using thiobarbituric acid [77]. Quinoa leaves were sampled from five plants per condition and immediately flash-frozen in liquid nitrogen for storage at −80 °C. Frozen leaf tissue was ground into a fine powder using a mortar and pestle, and 0.1 g of the pulverized sample was placed into a 2 mL microcentrifuge tube. One milliliter of 0.1% trichloroacetic acid (TCA) was added to the tube, followed by vigorous vortexing to ensure thorough mixing and homogenization. The samples were incubated on ice for 30 min and then centrifuged at 13,000 rpm for 15 min at 4 °C. The resulting supernatant was transferred to a fresh 2 mL microcentrifuge tube. Subsequently, 1.5 mL of a 20% TCA/0.5% thiobarbituric acid (TBA) solution was added, mixed well, and incubated at 95 °C for 15 min with the tubes partially capped. Following incubation, the tubes were rapidly cooled on ice, leading to the development of a reddish color. Finally, the samples were centrifuged at 10,000 rpm for 10 min at 4 °C, and the absorbance of the supernatant was measured at 532 nm and 600 nm to quantify the MDA concentration, which was expressed as mmol TBARS g^−1^ DW (dry weight), as described by Acuña-Rodriguez et al. ([78]).

#### 4.6.4. Proline Content Measurement

The quantification of proline content in quinoa leaves for each group was performed using the modified procedure of Bates et al. ([79]). Frozen quinoa leaf tissues (sampled from five different plants per condition) were ground into a fine powder, from which 100 mg of the pulverized material was transferred to a 2 mL centrifuge tube. Two milliliters of 3% sulfosalicylic acid were added, followed by thorough vortexing to ensure proper mixing and homogenization. The samples were centrifuged at 13,250 g for 15 min at 4 °C, after which the supernatant (approximately 450 µL) was carefully transferred to a new 2 mL centrifuge tube. Subsequently, 450 µL of ninhydrin reagent and 450 µL of glacial acetic acid were added to the supernatant, mixed thoroughly, and incubated at 100 °C for 45 min. After incubation, the tubes were rapidly cooled on ice for 30 min. To each tube, 1.5 mL of toluene was added; the mixture was vortexed to ensure homogeneity, and the tubes were centrifuged for 10 min at 13,250 g. Then, the toluene fraction was extracted, and its absorbance was measured at 520 nm using a spectrophotometer (Jenway 6300, Cole-Parmer, Staffordshire, UK) with L-proline as a standard for the calibration curve to determine proline concentration. Data were expressed in μmol g^−1^ DW (dry weight) tissue.

### 4.7. Molecular Markers: CqSOS1 and CqNHX1

To analyze the gene expression response of quinoa to salinity stress, specifically focusing on the salt extrusion mechanism, total RNA was extracted from 100 mg of leaf tissues of three plants per condition, using the E.Z.N.A. Plant RNA Kit, following the manufacturer’s instructions. The quality and concentration of the extracted RNA were checked using a NanoDrop spectrophotometer (ThermoFisher Scientific) and confirmed by agarose gel electrophoresis. Following RNA extraction, cDNA synthesis was performed using the Maxima H Minus First Strand cDNA Synthesis Kit according to the manufacturer’s instructions. The resulting cDNA was then used as a template for quantitative real-time PCR (qRT-PCR) to quantify transcript levels of *CqNHX1* and *CqSOS1A*, which encode proteins involved in salt extrusion and homeostasis [13]. The qRT-PCR was conducted using the Maxima SYBR Green/ROX qPCR Master Mix (2X) in a Magnetic Induction Cycler PCR (Biomolecular Systems). The qRT-PCR reaction was performed under the following conditions: an initial denaturation at 95 °C for 15 s, followed by 40 cycles of denaturation at 95 °C for 15 s, annealing at 55 °C for 30 s, and extension at 72 °C for 30 s [80]. A melt curve analysis was conducted at the end of the PCR run to verify the specificity of the amplification. Relative gene expression levels were calculated using the 2^−ΔΔCt^ method, and the housekeeping gene *CqGAPDH* was used as the internal control for normalization of gene expression data [13]. All reactions were performed in triplicate, and the results were analyzed to determine the effect of inoculation and salinity on the expression of *CqNHX1* and *CqSOS1A* in quinoa.

### 4.8. Elemental Composition Measurement

Spectral signatures of elements in quinoa leaf samples were obtained using a micro X-ray fluorescence spectrometer (µ-XRF) (M4 Tornado Plus (TM), Bruker, Bremen, Germany). Leaf samples were collected at the end of the experiment and directly analyzed using point measurements on the leaf surface. For each treatment, three leaves were collected from 12 independent plants (biological replicates), and three technical measurements were performed per leaf. Measurements were conducted using an accelerating voltage of 50 kV and a current of 30 μA, with a spot size of 160 μm and an exposure time of 20 ms per spot. Photons emitted from the samples were detected using an energy-dispersive silicon drift detector (SDD). Analyses were performed under vacuum conditions, which promote rapid dehydration of the leaf tissue during measurement and reduce potential interference from water in the X-ray signal. Elemental signals for Na, K and Cl were obtained using the multipoint measurement tool in the Bruker Esprit (TM) 1.2 software, which was also used for spectral processing and data extraction. Based on the μ-XRF signal intensities, the leaf K^+^/Na^+^ ratio was calculated as an indicator of ionic balance associated with salinity tolerance, a parameter deemed as a reliable predictor of salinity tolerance [81].

### 4.9. Statistical Analysis

To assess the effects of the experimental factors, genotype (Pandela or BO75) and fungal inoculation (inoculated or non-inoculated), statistical analyses were selected according to the distributions and characteristics of each measured variable. Plants’ survival rate, photochemical efficiency (Fv/Fm), MDA levels, proline concentration, and gene expression (*CqNHX1* and *CqSOS1A*) were analyzed using linear models, and the significance of factors was assessed using two-way Analysis of Variance (ANOVA) was employed to examine the main effects and potential interactions between both factors. When differences among groups were detected, post hoc comparisons were conducted using Tukey-adjusted estimated marginal means with statistical significance defined as *p*-value < 0.05. In the case of uXRF (elemental composition) measurements, the data failed to meet parametric assumptions; thus, we used the non-parametric Kruskal–Wallis test to identify significant differences across groups. When significant results were found, post hoc pairwise comparisons (Dunn’s multiple comparison tests) were conducted.

## Figures and Tables

**Table 1:** Molecular identification and taxonomic assignment of endophytic fungal isolates obtained from *Chenopodium quinoa* based on BLASTn analysis of the Internal Transcribed Spacer (ITS) rDNA region using the UNITE database as a reference.

Isolate Code	Closest UNITE Match	Accession Number	% Identity	Final Taxon Assigned
Isolate #1	*Alternaria chlamydosporigena*	KC584231	99.817	*Alternaria* sp. (section *Embellisia*)
Isolate #2	*Alternaria subcucurbitae*	KC584249	99.772	*Alternaria* sp. (section *Ulocladioides*)
Isolate #3	*Setophoma terrestris*	KF251246	99.802	*Setophoma* sp.

## Data Availability

The raw data supporting the conclusions of this article will be made available by the authors on request.

## Supplementary Materials

The following supporting information can be downloaded at: https://www.mdpi.com/article/10.3390/plants15152356/s1, Figure S1: Maximum-likelihood tree inferred from sequence alignments amongst *Alternaria* fungal species, based on DNA barcode ITS sequences (retrieved from UNITE; v10.0). Phylogenetic tree was constructed using IqTree3 and best-fit model (TIM3 + R2) was automatically selected by ModelFinder. *Alternaria longipes* was used as an outgroup. Numbers at nodes represent bootstrap support. Bold: *Alternaria* sp. sequences obtained from two different endophytic fungi isolated from *C. quinoa*; Figure S2: Maximum-likelihood tree inferred from sequence alignments amongst *Setophoma* fungal species, based on DNA barcode ITS sequences (retrieved from UNITE; v10.0). Phylogenetic tree was constructed using IqTree3 and best-fit model (TIM2 + F+I + G4) was automatically selected by ModelFinder. *Pseudopyrenochaeta lycopersici* was used as an outgroup. Numbers at nodes represent bootstrap support. Bold: Novel ITS sequence of a *Setophoma* sp. endophytic fungi isolated from *C. quinoa*. Table S1: Results of the two-way factorial ANOVA assessing the interactive effects of fungal endophyte inoculation (F), Genotype (G) and F x G of C. quinoa under 400 mM NaCl on plant survival, photochemical efficiency of PSII (Fv/Fm), lipid peroxidation, proline content, *NHX1* and *SOS1* relative expression. Table S2: Results of the non-parametric Kruskal–Wallis omnibus test for uXRF (estimated elemental composition) across four experimental groups, (inoculated and non-inoculated Pandela and BO75 C. quinoa plants, under 400 mM NaCl). Values represent the Chi-square (χ2) statistic and corresponding *p*-values. Table S3: Pairwise multiple comparisons of estimated elemental composition across genotypes and endophyte treatments. Pairwise comparisons were performed using Dunn’s post hoc test following an initial Kruskal–Wallis test. Significance codes: ns (non-significant), *: *p* < 0.05, **: *p* < 0.01.
