# Supplementary material for: Fungal Endophytes Are Associated with Improved Salinity Responses Across Quinoa Ecotypes
Source: Plants (Basel). 2026 Jul 30;15(15):2356. doi: 10.3390/plants15152356 (PMC13468198; doi:10.3390/plants15152356)
Supplement: Supplementary file 1 [file plants-15-02356-s001.zip › plants-4323296-supplementary.pdf]

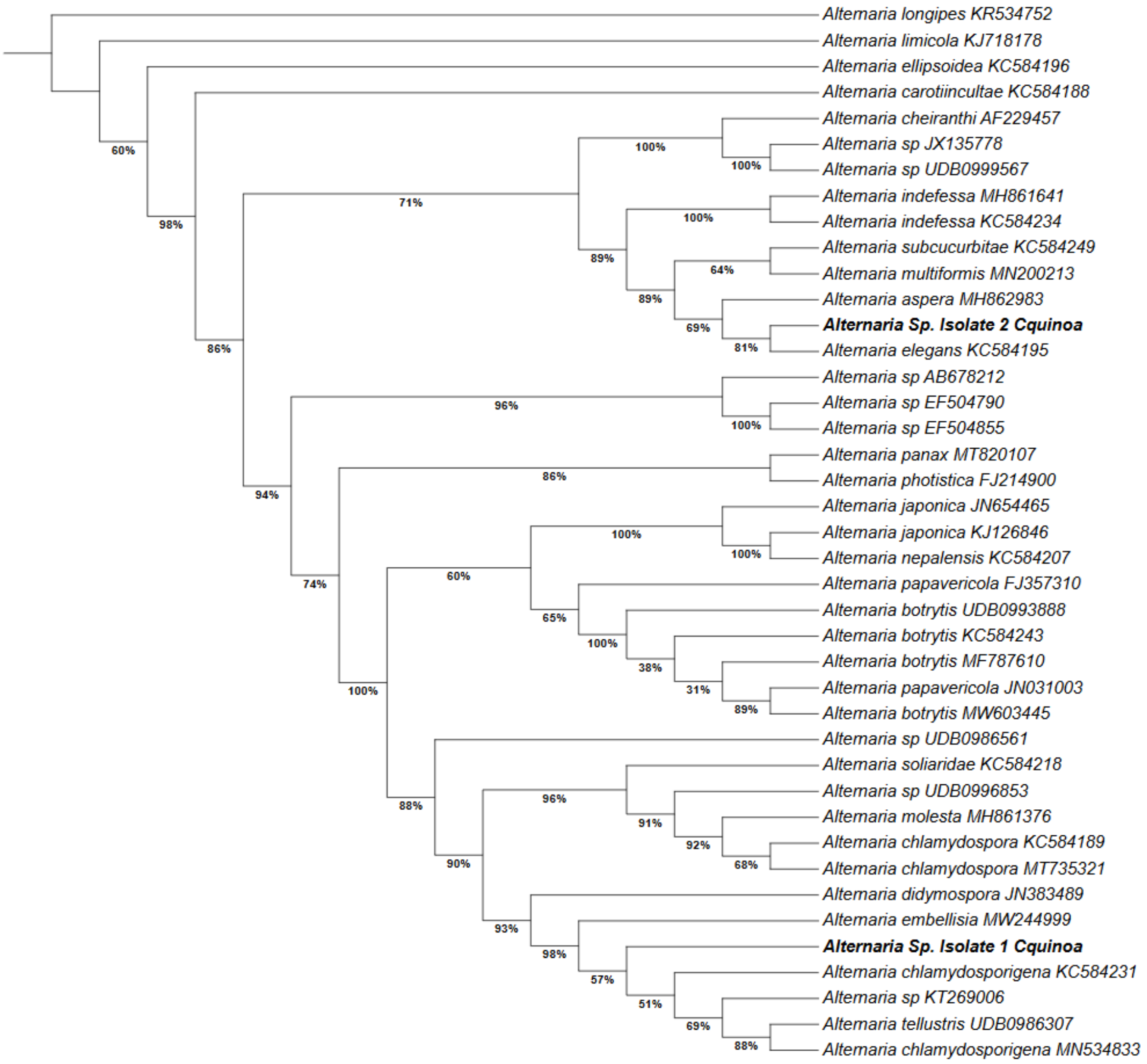


**Supplementary figure S1:** Maximum-likelihood tree inferred from sequence alignments amongst *Alternaria* fungal species, based on DNA barcode ITS sequences (retrieved from UNITE; v10.0). Phylogenetic tree was constructed using IqTree3 and best-fit model (TIM3+R2) was automatically selected by ModelFinder. *Alternaria longipes* was used as an outgroup. Numbers at nodes represent bootstrap support. Bold: *Alternaria* sp. sequences obtained from two different endophytic fungi isolated from *C. quinoa*

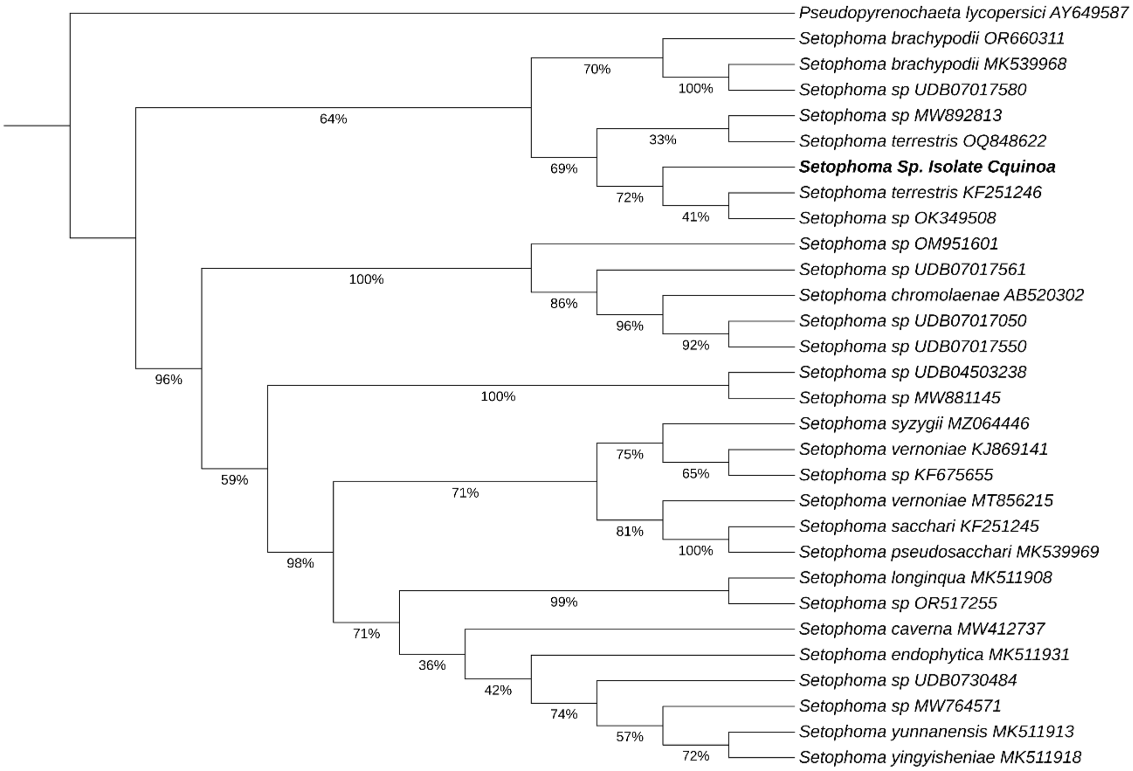


**Supplementary figure S2:** Maximum-likelihood tree inferred from sequence alignments amongst *Setophoma* fungal species, based on DNA barcode ITS sequences (retrieved from UNITE; v10.0). Phylogenetic tree was constructed using IqTree3 and best-fit model (TIM2+F+I+G4) was automatically selected by ModelFinder. *Pseudopyrenochaeta lycopersici* was used as an outgroup. Numbers at nodes represent bootstrap support. Bold: Novel ITS sequence of a *Setophoma* sp. endophytic fungi isolated from *C. quinoa*

**Supplementary table S1.** Results of the two-way factorial ANOVA assessing the interactive effects of fungal endophyte inoculation (F), Genotype (G) and F x G of *C. quinoa* under 400 mM NaCl on plant survival, photochemical efficiency of PSII (Fv/Fm), lipid peroxidation, proline content, *NHX1* and *SOS1* relative expression.

| Source of Variation | df | Mean Sq | F value | P-value | Significance |
|---|---|---|---|---|---|
| **Survival** | | | | | |
| Fungal endophyte (F) | 1 | 0.6886 | 4.984 | 0.0275 | * |
| Genotype (G) | 1 | 6.035 | 43.68 | < 0.0001 | *** |
| F x G | 1 | 0.1993 | 1.442 | 0.2322 | |
| Residuals | 116 | 0.1382 | | | |
| | | | | | |
| **Photochemical efficiency** | | | | | |
| Fungal endophyte (F) | 1 | 0.019 | 42.31 | < 0.001 | *** |
| Genotype (G) | 1 | 0.029 | 63.36 | < 0.001 | *** |
| F x G | 1 | 0.018 | 40.43 | < 0.001 | *** |
| Residuals | 36 | 0.016 | | | |
| | | | | | |
| **Lipid Peroxidation** (MDA) | | | | | |
| Fungal endophyte (F) | 1 | 38.35 | 38.79 | < 0.0001 | **** |
| Genotype (G) | 1 | 14.66 | 14.83 | 0.0014 | ** |
| F x G | 1 | 53.40 | 54.01 | < 0.0001 | **** |
| Residuals | 16 | 0.9987 | | | |
| | | | | | |
| **Proline Content** | | | | | |
| Fungal endophyte (F) | 1 | 183024 | 66.55 | < 0.001 | **** |
| Genotype (G) | 1 | 25609 | 9.311 | 0.0076 | ** |
| F x G | 1 | 13.05 | 0.004 | 0.9459 | |
| Residuals | 16 | 2759 | | | |
| | | | | | |
| ***NHX1* expression** | | | | | |
| Fungal endophyte (F) | 1 | 0.173 | 0.7691 | 0.3909 | |
| Genotype (G) | 1 | 1.215 | 5.389 | 0.0309 | * |
| F x G | 1 | 1.162 | 5.152 | 0.0344 | * |
| Residuals | 20 | 4.509 | | | |
| | | | | | |
| ***SOS1* expression** | | | | | |
| Fungal endophyte (F) | 1 | 0.405 | 2.566 | 0.1249 | |
| Genotype (G) | 1 | 1.270 | 8.032 | 0.0103 | * |
| F x G | 1 | 1.325 | 8.385 | 0.0089 | ** |
| Residuals | 20 | 3.162 | | | |

**Significance codes: *: p < 0.05, **: p < 0.01, ***: p < 0.001, ****: p < 0.0001**

**Supplementary table S2.** Results of the non-parametric Kruskal-Wallis omnibus test for uXRF (estimated elemental composition) across four experimental groups, (inoculated and non-inoculated Pandela and BO75 *C. quinoa* plants, under 400 mM NaCl). Values represent the Chi-square ($\chi^2$) statistic and corresponding *p*-values.

| **Kruskal-Wallis test** | **K+** | **Cl-** | **Na+** | **K/Na ratio** |
|---|---|---|---|---|
| **Chi-square ($\chi^2$)** | 17,3 | 3,96 | 23,58 | 18,55 |
| *p*-value | 0,0006 | 0.2658 | <0.0001 | 0,0003 |
| *p*-value summary | *** | ns | **** | *** |
| Significant differences across groups ($p < 0.05$)? | Yes | No | Yes | Yes |
| Number of groups | 4 | 4 | 4 | 4 |
| df | 3 | 3 | 3 | 3 |
| Data summary | | | | |
| Number of treatments (columns) | 4 | 4 | 4 | 4 |
| Number of values (total) | 48 | 48 | 48 | 48 |

**Significance codes: ns (non-significant), *: p < 0.05, **: p < 0.01, ***: p < 0.001**

**Supplementary table S3**. Pairwise multiple comparisons of estimated elemental composition across genotypes and endophyte treatments. Pairwise comparisons were performed using Dunn's post-hoc test following an initial Kruskal-Wallis test. Significance codes: ns (non-significant), *: $p < 0.05$, **: $p < 0.01$

| Element | Comparison (Group 1 vs. Group 2) | Rank Sum Difference | Z-statistic | Adjusted p-value | Significance |
|---|---|---|---|---|---|
| **Na+** | Pandela E- vs. Pandela E+ | 0.417 | 0.073 | >0.9999 | ns |
| | Pandela E- vs. BO75 E- | -20.25 | 3.543 | 0.0024 | ** |
| | Pandela E- vs. BO75 E+ | -18.5 | 3.237 | 0.0073 | ** |
| | Pandela E+ vs. BO75 E- | -20.667 | 3.616 | 0.0018 | ** |
| | Pandela E+ vs. BO75 E+ | -18.917 | 3.31 | 0.0056 | ** |
| | BO75 E- vs. BO75 E+ | 1.75 | 0.306 | >0.9999 | ns |
| **K+** | Pandela E- vs. Pandela E+ | -6.333 | 1.108 | >0.9999 | ns |
| | Pandela E- vs. BO75 E- | 12.25 | 2.143 | 0.1925 | ns |
| | Pandela E- vs. BO75 E+ | 13.75 | 2.406 | 0.0968 | ns |
| | Pandela E+ vs. BO75 E- | 18.583 | 3.251 | 0.0069 | ** |
| | Pandela E+ vs. BO75 E+ | 20.083 | 3.514 | 0.0026 | ** |
| | BO75 E- vs. BO75 E+ | 1.5 | 0.262 | >0.9999 | ns |
| **K/Na ratio** | Pandela E- vs. Pandela E+ | -4.583 | 0.802 | >0.9999 | ns |
| | Pandela E- vs. BO75 E- | 14.25 | 2.493 | 0.076 | ns |
| | Pandela E- vs. BO75 E+ | 15.333 | 2.683 | 0.0438 | * |
| | Pandela E+ vs. BO75 E- | 18.833 | 3.295 | 0.0059 | ** |
| | Pandela E+ vs. BO75 E+ | 19.917 | 3.485 | 0.003 | ** |
| | BO75 E- vs. BO75 E+ | 1.083 | 0.19 | >0.9999 | ns |
| **Cl-** | Pandela E- vs. Pandela E+ | -2.083 | 0.365 | >0.9999 | ns |
| | Pandela E- vs. BO75 E- | 8.5 | 1.487 | 0.8218 | ns |
| | Pandela E- vs. BO75 E+ | 0.583 | 0.102 | >0.9999 | ns |
| | Pandela E+ vs. BO75 E- | 10.583 | 1.852 | 0.3844 | ns |
| | Pandela E+ vs. BO75 E+ | 2.667 | 0.467 | >0.9999 | ns |
| | BO75 E- vs. BO75 E+ | -7.917 | 1.385 | 0.9961 | ns |
